# Conductive Polymer-Based Interlayers in Restraining the Polysulfide Shuttle of Lithium–Sulfur Batteries

**DOI:** 10.3390/molecules29051164

**Published:** 2024-03-05

**Authors:** Xincheng Hu, Xiaoshuang Zhu, Zhongshuai Ran, Shenghao Liu, Yongya Zhang, Hua Wang, Wei Wei

**Affiliations:** 1 Henan Engineering Center of New Energy Battery Materials, College of Chemistry and Chemical Engineering, Shangqiu Normal University, Shangqiu 476000, China; huxincheng2019@163.com (X.H.);; 2 Center of Green Catalysis, College of Chemistry, Zhengzhou University, Zhengzhou 450001, China; 3 School of Chemistry, Beijing Advanced Innovation Center for Biomedical Engineering, Beihang University, Beijing 100191, China

**Keywords:** lithium–sulfur batteries, conductive polymer, interlayers, shuttle effects

## Abstract

Lithium–sulfur batteries (LSBs) are considered a promising candidate for next-generation energy storage devices due to the advantages of high theoretical specific capacity, abundant resources and being environmentally friendly. However, the severe shuttle effect of polysulfides causes the low utilization of active substances and rapid capacity fading, thus seriously limiting their practical application. The introduction of conductive polymer-based interlayers between cathodes and separators is considered to be an effective method to solve this problem because they can largely confine, anchor and convert the soluble polysulfides. In this review, the recent progress of conductive polymer-based interlayers used in LSBs is summarized, including free-standing conductive polymer-based interlayers, conductive polymer-based interlayer modified separators and conductive polymer-based interlayer modified sulfur electrodes. Furthermore, some suggestions on rational design and preparation of conductive polymer-based interlayers are put forward to highlight the future development of LSBs.

## 1. Introduction

Lithium–sulfur batteries (LSBs) are considered one of the most promising energy storage devices due to the advantages of high theoretical specific capacity, resource abundance and low toxicity. However, the severe diffusion of polysulfides during repeated charging/discharging leads to the low utilization of active materials, corrosion of lithium anode and large polarization of sulfur cathode, and seriously limits their practical application [1,2,3,4,5,6,7,8]. In this regard, plenty of work has been carried out to suppress the diffusion of polysulfides in LSBs [9,10,11,12,13,14], such as the design of host materials [4], the introduction of interlayers between cathodes and separators [15,16,17,18,19] and the optimization of electrolytes [20,21]. Specifically, the introduction of interlayers between cathodes and separators is considered a promising approach because it can obviously suppress the shuttle of lithium polysulfides, and the preparation method is simple and easy to scale-up [22,23]. In the past few years, pure carbon materials [15], pure conductive polymers [24], metal foam foil [16], metal compounds [25,26,27], carbon-based materials [28,29] and conductive polymer-based materials [30,31] have been employed as interlayers by scientists. Among them, conductive polymers have more advantages of excellent conductivity, strong polysulfide adsorption capacity, moderate mechanical strength and porous structure [31,32]. In addition, some conductive polymers are electrochemically active and can contribute capacity to offset the reduction of gravimetric energy density caused by the addition of interlayers [33]. In 2015, Ma et al. first inserted polypyrrole (PPy) nanotube film between the cathode and the separator. The first discharge capacity of the as-assembled LSB could reach 1102 mAh g^−1^, and a high retention rate of 64% was obtained after 300 cycles at 0.5 C [24]. Moon et al. synthesized a highly uniform polyaniline (PANI) film on the sulfur cathode by a transfer printing method. The battery with the printed PANI layer showed a high capacity retention of 96.4% after 200 cycles at 1 C [34]. Although achievements have been made during the past few years, there is still no special report on the research progress of conductive polymer-based interlayers.

In this review, the recent progress of commonly used conductive polymer-based interlayers in LSBs is systematically summarized, including free-standing conductive polymer-based interlayers, conductive polymer-based interlayer modified separators and conductive polymer-based interlayer modified sulfur electrodes. In addition, research progress and future development of conductive polymer-based interlayers are summarized and prospected.

## 2. Current Designing and Working Principles of Interlayers in LSBs

**Figure 1:**
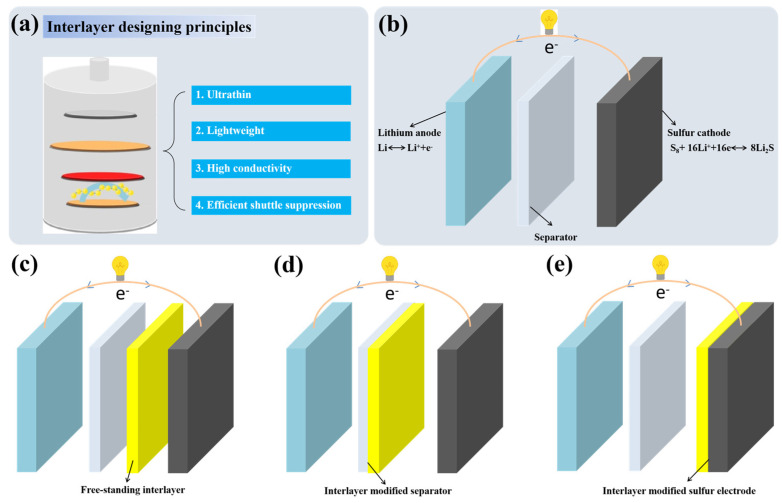
(**a**) Schematic of the current design principles for interlayers in LSBs. (**b**) Schematic representations of LSBs. (**c**) LSBs inserted with free−standing conductive polymer−based interlayers. (**d**) LSBs with conductive polymer−based interlayer modified separators. (**e**) LSBs with conductive polymer−based interlayer modified sulfur electrodes.

As shown in Figure 1a, interlayers between electrolytes and sulfur cathodes play an important role in mitigating the shuttle effect of polysulfides. Except for the efficient suppression of the shuttle effect, ultrathin and lightweight characteristics are necessary for interlayers to ensure the energy density of the full cell. The possession of both electronic conductivity and ionic conductivity simultaneously are also essential for interlayers to transport Li^+^ and electronics [9,24]. Based on these designing principles of interlayers in LSBs, conductive polymers that are functional and lightweight and have easy processibility and high ionic/electronic conductivity should be one of the most promising materials for the construction of interlayers for LSBs. The preparation methods mainly include vacuum filtration, blade scraping/coating, in situ vapor-phase polymerization, binder-free coating, spray coating, splash coating and oxidation polymerization. 

The working principle of LSBs is shown in Figure 1b. During the discharge process, the negative lithium electrode loses electrons to form lithium ions, while the positive sulfur electrode receives electrons transmitted through the external circuit and combines with the lithium ions to generate Li_2_S. The charging process is the opposite. In fact, during the charging and discharging process, the positive electrode of LSBs undergoes a complex multi-electron reaction process. During discharging, the cyclic S_8_ molecules first open the ring and combine with Li^+^ to form soluble lithium polysulfides (Li_2_S_x_, 4 ≤ x ≤ 8), which are then further reduced to form solid Li_2_S_2_ and Li_2_S. The intermediate lithium polysulfides are easily soluble in the electrolyte and shuttle between the positive and negative electrodes, resulting in the so-called “shuttle effect”, which leads to the loss of active substances in the positive and negative electrodes, battery capacity degradation and a decrease in coulombic efficiency [18,35]. Inserting conductive polymer-based interlayers between electrolytes and sulfur cathodes, including free-standing conductive polymer-based interlayers (Figure 1c), conductive polymer-based interlayer modified separators (Figure 1d) and conductive polymer-based interlayer modified sulfur cathodes (Figure 1e), has been widely developed and proved to be a promising strategy. The conductive polymers Poly(3,4-ethylenedioxythiophene)(PEDOT), PANI and PPy used in this article are generally proton-doped, so protons can tightly connect the polymer with polysulfide anions through hydrogen bonds. During charging/discharging, the hydrogen bond between the proton-doped polymer and lithium polysulfide can make the polymer more effectively adsorb polysulfides [24,36,37].

## 3. Free-Standing Conductive Polymer-Based Interlayers

Functional interlayers inserted between separator and cathode can immobilize the soluble polysulfides and efficiently inhibit the shuttle effect. Free-standing interlayers usually exhibit a stronger adsorption capacity for polysulfides, and the certain thickness allows them to support themselves. Conductive polymers with imine or conjugated structures, mainly including PPy, PANI and PEDOT, can greatly chemisorb polysulfides and limit polysulfide diffusion [35]. Inserting a free-standing conductive polymer-based interlayer between the sulfur electrode and the separator can improve the electrochemical performance of LSBs. When the free-standing conductive polymer-based interlayer is assembled into LSBs, the shuttle of polysulfides is inhibited, Li^+^ migration is selectively allowed to pass through, the redox kinetics of polysulfides are promoted and, finally, the utilization rate and cycle performance of active materials are improved. Moreover, inserting a free-standing conductive polymer-based interlayer between the separator and the cathode can improve the conductivity of the electrodes and effectively inhibit the diffusion of polysulfides [18,38,39,40,41,42].

### 3.1. Free-Standing PPy-Based Interlayers

**Figure 2:**
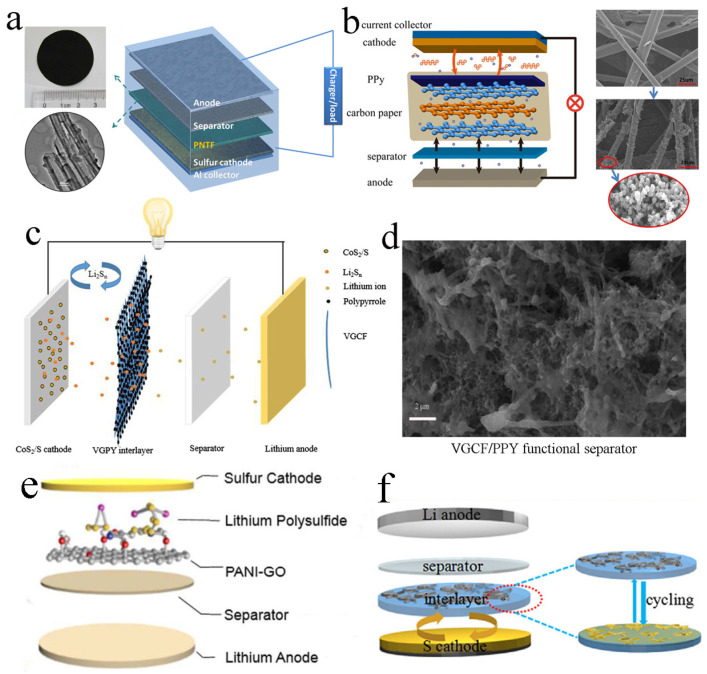
(**a**) Image of the PPy nanotube film, TEM image of PPy nanotubes and schematic cell configuration of LSBs. Reproduced from ref. [24] with permission from Elsevier (Amsterdam, The Netherlands), copyright 2015. (**b**) Schematic configuration of an LSB with PPy interlayer inserted between the separator and sulfur cathode. Reproduced from ref. [43] with permission from The Royal Society of Chemistry (London, UK), copyright 2015. (**c**) Configuration of LSB with the bifunctional PPy-coated vapor-grown carbon fiber interlayer between the CoS_2_/S cathode and PP separator. Reproduced from ref. [44] with permission from Springer (Berlin/Heidelberg, Germany), copyright 2019. (**d**) SEM image of vapor-grown carbon fiber/PPY functional separator. Reproduced from ref. [45] with permission from Elsevier, copyright 2019. (**e**) Schematic illustration of a PANI-GO interlayer inserted between the separator and sulfur cathode in LSBs. Reproduced from ref. [46] with permission from The Royal Society of Chemistry, copyright 2018. (**f**) Schematic configuration of rechargeable LSBs with PEDOT: PSS-CNT interlayer. Reproduced from ref. [36] with permission from WILLY-VCH (Hoboken, NJ, USA), copyright 2017.

The first reported free-standing PPy interlayer was in 2015. Ma et al. prepared a free-standing PPy nanotube film (Figure 2a). This novel PPy nanotube film had a high specific surface area and a large number of pores, which could achieve the effect of enhancing sulfur utilization and capacity. By using the PPy interlayer, the interfacial polarization could be reduced, the serious shuttle effect in the cycle could be significantly inhibited and the sulfur-containing active materials could be rearranged. Appling the interlayer to LSBs for testing, the initial discharge capacity reached a high value of 1102 mA h g^−1^. At the 0.5 C rate of 300 cycles, the capacity rate was also high [24].

In addition to pure PPy as the free-standing interlayer, PPy-modified carbon paper has also been used as the interlayer in LSBs. Wu et al. prepared an interlayer composed of PPy-treated carbon paper (Figure 2b). This interlayer promoted the efficiency of charge transfer on the electrode surface, improved the utilization of sulfur and greatly inhibited the shuttle of polysulfides. Due to the good conductivity of the material and characteristic of many voids, the battery composed of PPy coated with carbon paper presented better cycle stability and electrochemical performance. After 200 cycles at 0.5 C, the battery could still retain a capacity of 555 mA h^−1^ [43].

The free-standing interlayer of carbon paper coated with PPy has also been prepared. This interlayer had the following two functions: it effectively captured polysulfides and greatly reduced the polarization of the sulfur cathode. Deng and co-workers prepared a vapor-grown carbon fiber/PPy interlayer (Figure 2c). The vapor-grown carbon fiber could improve the electronic conductivity of the material, and the PPy could adsorb and hinder the diffusion of polysulfides. The initial discharge-specific capacity of the assembled battery was up to 1085 mA h g^−1^ at 0.1 C discharge. After 200 cycles, the capacity was still as high as 768 mAh g^−1^, and the capacity decay rate per cycle was only 0.146% [44]. Moreover, Li et al. prepared a bifunctional interlayer composed of PPy-coated vapor-grown carbon fiber to capture polysulfides in LSBs (Figure 2d). The as-prepared bifunctional PPy-coated vapor-grown carbon fiber interlayer could physically adsorb the polysulfides and simultaneously reduce the polarization of the sulfur cathode. Compared with the LSB without interlayers, the LSB with the bifunctional PPy-coated vapor-grown carbon fiber interlayer exhibited excellent electrochemical performance, with an initial discharge capacity of 1204.51 mA h g^−1^ and a capacity retention of 1262.56 mA h g^−1^ at 0.1 C after 300 cycles [45].

### 3.2. Free-Standing PANI-Based Interlayers

A free-standing PANI-based interlayer has been reported to promote the development of LSBs. For instance, a free-standing PANI-GO interlayer was inserted between the separator and the sulfur cathode by Yin and co-workers to suppress the diffusion of polysulfides (Figure 2e). Equipped with this interlayer, the as-assembled LSB displayed a relatively high initial discharge capacity of 1261 mA h g^−1^ and a capacity retention rate of 73.0% at 0.5 C after the 150th cycling [46].

### 3.3. Free-Standing PEDOT: Poly(styrene sulfonate)(PSS)-Based Interlayers

Conductive PEDOT: PSS is also used to prepare a free-standing interlayer to improve the energy density and rate performance of the LSBs. A PEDOT: PSS-CNT interlayer with the feature of highly conductive and lightweight was fabricated and integrated into LSBs by Wang and co-workers (Figure 2f). PEDOT: PSS acted as the polysulfide reservoir soft buffer to suppress the migration of polysulfides, and CNTs provided porous transport pathways for the rapid Li^+^ diffusion. As a result, the free-standing composite interlayer greatly hindered the movement of polysulfides to lithium anode through chemical interaction. The LSB assembled with the PEDOT: PSS-CNT interlayer exhibited excellent cycle stability. After 200 cycles of charging and discharging at 0.5 C, the LSB still retained a specific capacity of 653 mA h g^−1^ [36].

## 4. Conductive Polymer-Based Interlayer Modified Separators

A free-standing interlayer normally needs a certain thickness to allow it to support itself, which reduces the gravimetric energy density, and the solid–solid interfacial contact between interlayer and separator/sulfur cathode is insufficient. Separators, mainly composed of polymer films with many voids, are one of the most important parts of LSBs to prevent direct contact and short circuit between the cathode and anode. However, the commonly used polymer film has no functional groups to select ions. Soluble polysulfides can easily pass through the voids of the separators to reach the lithium anode, causing a large loss of active materials [47,48]. Conductive polymer-based interlayers with imine or conjugated structures can greatly chemisorb polysulfides and limit their diffusion [35]. If the separator is modified with conductive polymer-based materials, soluble polysulfides can be effectively captured, fixed and converted, and thus improve the cycling performance of the battery (or reduce the shuttle effects of polysulfides) [49,50]. 

### 4.1. PPy-Based Material Modified Separators

**Figure 3:**
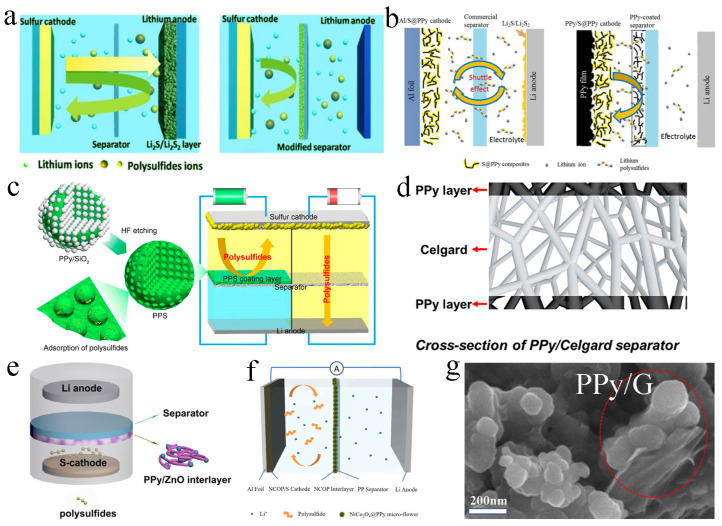
(**a**) Schematic representation of the design of a separator in LSBs configurations, and the comparison of batteries with the common separator and a PPy-modified separator. Reproduced from ref. [53] with permission from The Royal Society of Chemistry, copyright 2016. (**b**) Comparison of LSBs with the traditional separator and PPy-coated separator. Reproduced from ref. [33] with permission from Elsevier, copyright 2018. (**c**) Schematic diagram of LSBs equipped with porous polypyrrole sphere-modified separator. Reproduced from ref. [54] with permission from MDPI (Basel, Switzerland), copyright 2019. (**d**) Graphic illustration of LSBs with a PPy-modified separator. Reproduced from ref. [55] with permission from Elsevier, copyright 2019. (**e**) The suppression of polysulfides with PPy/ZnO interlayer modified separator. Reproduced from ref. [57] with permission from Springer, copyright 2018. (**f**) Schematic of cell configuration for NiCo_2_O_4_@PPy/S cathode with NiCo_2_O_4_@PPy interlayer. Reproduced from ref. [58] with permission from Elsevier, copyright 2022. (**g**) High-resolution scanning electron microscope (SEM) image of PPy/graphene composite. Reproduced from ref. [59] with permission from WILLY-VCH, copyright 2021.

PPy has good affinity with electrolytes and strong adsorption capacity for polysulfides and can also contribute to the capacity. These outstanding advantages of PPy enable it to be extensively used in the modification of separators in LSBs [51,52]. Ma et al. used PPy nanotubes, PPy nanowires and reduced graphene oxide (rGO) to modify the separators (Figure 3a). They found that the diffusion of polysulfides was inhibited and the utilization of sulfur cathode was improved. Attributed to the excellent affinity of PPy with lithium polysulfides and electrolytes, the PPy-modified separators showed enhanced electrochemical performance in LSBs. After 200 cycles at 0.2 C, the discharge capacity of the LSB without separator modification was only 215 mA h g^−1^ and the coulomb efficiency was less than 80%. In contrast, the discharge capacity of the batteries with PPy nanotubes and PPy nanowire modified separators was up to 865.4 mA h g^−1^ and 832.1 mA h g^−1^, with the corresponding coulomb efficiency of 89.4% and 88.1%, respectively [53]. In addition, Li et al. designed a PPy nanofiber-coated separator and assembled it into flexible LSBs (Figure 3b). The PPy nanofiber-coated separator could successfully suppress the diffusion of soluble polysulfides, and could also be used as an upper collector. The kinetic speed of the electrochemical reaction was significantly accelerated. PPy had electrochemical activity, which could provide part of the capacity. Combined with the improved sulfur cathode, the initial specific capacity of the LSB was as high as 1064 mA h g^−1^, and after the 20th charge/discharge cycle at 0.1 C, the capacity could still be maintained at a high value of 848 mA h g^−1^ [33].

In addition to PPy nanotubes, PPy nanowires and PPy nanofibers, PPy spheres have also been applied to modify the separators. Li et al. successfully prepared a new layered porous PPy sphere to modify the separator (Figure 3c). When PPy sphere slurry was coated on the separator, the polarization of the cathode could be significantly reduced, and polysulfides could be effectively suppressed, thus significantly improving the sulfur utilization rate. The modified separator was assembled into LSBs. After 100 cycles at 0.2 C, the capacity was 855 mA h g^−1^, and the capacity was 507 mA h g^−1^ after discharge at 3 C [54].

Other than coating PPy on one side of the separator close to the cathode, Li et al. coated PPy on both surfaces of the commercially available separator by in situ gas phase polymerization (Figure 3d). By using this separator, electrolyte absorption was enhanced, and the shuttle effect was inhibited, thus improving electrochemical performance. With this double-sided coating separator, the capacity decay rate was only 0.083% per circle after stable circulation for 250 cycles at 0.5 C. The cycle performance was also excellent at higher area capacity (4.8 mA h cm^−2^); after 150 cycles at 0.2 C, the capacity retention rate kept up to 75.6% [55]. Similarly, Fu et al. also fabricated a dual-functional separator with PPy coating on both surfaces by a vapor-phase polymerization method, which inhibited the shuttle of polysulfides. The separator was assembled into LSBs with excellent electrochemical performance; for example, the decay rate of 500 charge/discharges at 1 A g^−1^ was only 0.037% [56].

To enhance the chemisorptive binding with polysulfides, metal oxides have also been adopted to combine with PPy in the modified separator materials to further chemisorb polysulfides. For instance, Yin et al. prepared a PPy/ZnO modified separator to anchor polysulfides (Figure 3e). Due to the existence of ZnO and PPy, the PPy/ZnO interlayer could prevent the migration of soluble polysulfides and greatly improve the performance of LSBs. After charging/discharging for 100 cycles at 0.2 C, the battery retained a high specific capacity of 579 mA h g^−1^ [57]. Moreover, Ma et al. synthesized a NiCo_2_O_4_@PPy multifunctional interlayer (Figure 3f). Flowery NiCo_2_O_4_ and PPy could physically adsorb, chemically anchor and further accelerate the transformation of polysulfides. The initial capacity of the battery could reach 1588 mA h g^−1^ at 0.1 C discharge, and the average coulomb efficiency was 95.2%. At 2 C, the discharge-specific capacity was also up to 740 mA h g^−1^. After 400 cycles of charging and discharging, a capacity decay rate of 0.107% could be obtained [58].

Besides chemical anchor of polysulfides by combining metallic oxides with PPy, physical confinement by carbon materials with PPy can also be used to capture polysulfides. Zhou et al. developed a PPy/graphene composite interlayer (Figure 3g), which could inhibit the dissolution of polysulfides and improve the conversion and utilization of sulfur cathode. As a result, the initial specific capacity of LSBs was as high as 1443 mA h g^−1^. When charged and discharged 1000 times, it could maintain 0.04% capacity decay rate per cycle at 1 C [59].

### 4.2. PANI-Based Material Modified Separators

**Figure 4:**
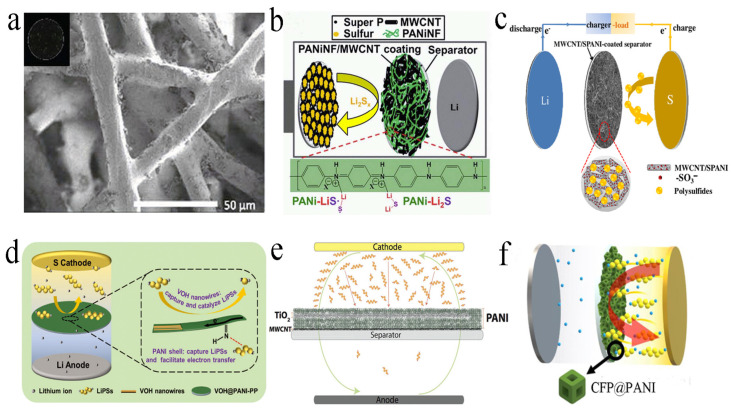
(**a**) SEM images and an inset photograph of hydrolyzed polyethylene−PANI. Reproduced from ref. [64] with permission from The Royal Society of Chemistry, copyright 2019. (**b**) Graphic illustration of the interception and immobilization of polysulfides by PANI nanofibers/MWCNT−functionalized separator. Reproduced from ref. [65] with permission from The Royal Society of Chemistry, copyright 2015. (**c**) Schematic configuration of LSB with MWCNT/sulfonated polyaniline−coated separator. Reproduced from ref. [66] with permission from Elsevier, copyright 2018. (**d**) Schematic illustration of the mechanism of high−performance LSBs equipped with V_2_O_5_·nH_2_O@PANI−PP separator. Reproduced from ref. [67] with permission from WILLY-VCH, copyright 2021. (**e**) Schematic presentation of the coating structure of a laminated separator that illustrates the polysulfide flux diagram during the charge/discharge of the LSBs. Reproduced from ref. [68] with permission from WILLY-VCH, copyright 2016. (**f**) Schematic configuration of LSB with Co−Fe Prussian blue analogue@PANI−coated separator. Reproduced from ref. [69] with permission from ACS Publications (Washington, DC, USA), copyright 2021.

PANI has the characteristics of high ionic conductivity and a large void ratio, which could be employed to modify separators [60,61,62]. The modified separator could suppress the shuttle of polysulfides and reduce the surface impedance of the separator, while the inherent pore structure of the separator was barely affected [51,63]. For example, Tubtimkuna et al. presented hydrolyzed polyethylene grafted PANI via amide coupling reaction as a modified separator (Figure 4a). This separator could effectively inhibit the diffusion of polysulfides through the amine group of PANI. This separator not only improved the specific capacity, but also inhibited the capacity attenuation [64].

In contrast to pure PANI, combining carbon materials with PANI as interlayers can further capture polysulfides. Chang et al. prepared a modified separator composed of PANI nanofibers/MWCNT (Figure 4b). The PANI coating could be tightly combined on the commercial separator, which was highly effective in capturing the diffused polysulfides. This was mainly attributed to the strong chemical interaction of sulfur-containing species and the imine group (–N=C) from the quinone ring. When the modified separator was assembled into a battery, the initial discharge capacity reached 1020, 867 and 791 mA h g^−1^ at 0.2 C, 0.5 C and 1 C, respectively [65]. Shi and co-workers prepared a separator modified by MWCNT/PANI sulfide (Figure 4c), which could selectively control the migration of Li^+^ and inhibit the diffusion and shuttle of polysulfides. Therefore, even at high sulfur loading (5 mg cm^−2^), the LSBs assembled by this separator still exhibited excellent electrochemical performance, and the reversible capacity was up to 1078 mA h g^−1^, which was better than the performance of a pure separator and an MWCNT modified separator [66]. 

In addition to physical absorption of polysulfides by carbon materials, a chemical anchor was also used to capture polysulfides. Chen et al. modified amorphous vanadium pentoxide (V_2_O_5_) nanowires encapsulated in PANI onto commercial polypropylene separators (Figure 4d). When the separator was assembled into LSBs, it had higher electrochemical performance than the single-component modified separator. The adsorption test showed that the adsorption capacity of V_2_O_5_∙nH_2_O@PANi towards Li_2_S_6_ was stronger, mainly due to the positive synergy between V_2_O_5_∙nH_2_O and PANI [67]. To physically absorb and chemically anchor the polysulfides, Archer and collaborators applied Langmuir–Blodgett scooping coating technology to prepare an MWCNT/TiO_2_ nanoparticle/PANI interlayer (Figure 4e). MWCNT physically blocks polysulfides, while PANI and TiO_2_ nanoparticles can chemically adsorb polysulfides. Under different discharge rates, the battery can also show higher capacity and better cycle performance [68]. Besides physical absorption and/or chemical anchor of polysulfides, the catalytic effect of interlayer can be largely improved. Jo et al. reported a hollow Co-Fe Prussian blue analogue encapsulated by PANI, and coating PANI on the prepared hollow Co-Fe Prussian blue could improve the catalytic effect and enhance the conductivity (Figure 4f). After 100 charging/discharging cycles at 1 A g^−1^, the capacity retention rate was as high as 83.5%, with an average coulomb efficiency of 99.5% [69].

### 4.3. PEDOT: PSS-Based Material Modified Separators

**Figure 5:**
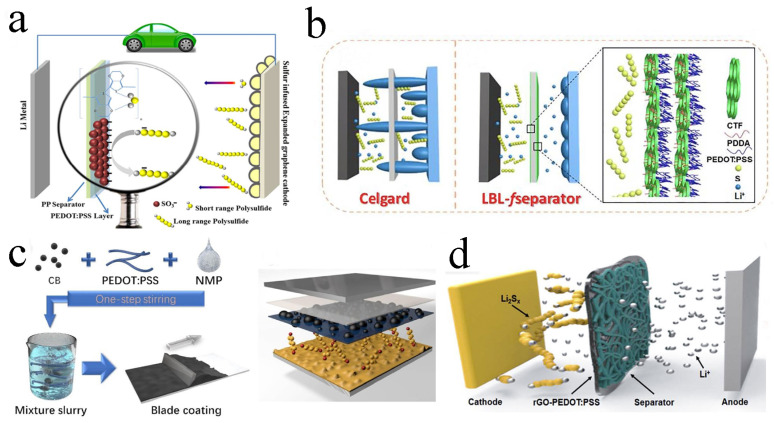
(**a**) Diagrammatic representation of an LSB featuring a PEDOT: PSS−coated separator and a sulfur-infused expanded graphene cathode. Reproduced from ref. [76] with permission from The Royal Society of Chemistry, copyright 2016. (**b**) Schematic diagrams of an LSB with the pristine Celgard and layer−by−layer−*f*separator. Reproduced from ref. [77] with permission from Elsevier, copyright 2021. (**c**) The preparation process of carbon black/PEDOT: PSS−modified separator. Reproduced from ref. [78] with permission from WILLY−VCH, copyright 2019. (**d**) Schematic battery configuration of an LSB with rGO−PEDOT: PSS−coated separator. Reproduced from ref. [79] with permission from ACS, copyright 2018.

PEDOT is a polythiophene derivative with high conductivity, excellent chemical stability and flexibility, which can inhibit the shuttle of polysulfides and accelerate the electron transfer speed, further improving the energy density and cycle stability of LSBs [70,71,72]. Within separators modified by PEDOT, the electrochemical performance of LSBs can be significantly improved [51,73,74,75]. For example, Abbas et al. reported a separator that was mainly composed of PEDOT: PSS sprayed onto the commercial separator (Figure 5a). The negatively charged SO_3_^−^ in PSS could electrostatically shield the soluble polysulfides, and the O, S atoms in PEDOT could chemically interact with low-order polysulfides to prevent them from passing through the separator. When the LSB was assembled and charging/discharging was repeated 1000 times at 0.25 C, the attenuation rate was as low as 0.0364% per cycle [76]. With the help of electrostatic layer-by-layer self-assembly strategy, Shi and co-workers integrated PEDOT: PSS and a functional covalent triazine framework (CTF) to develop a layer-by-layer *f*separator. Functional CTF with high porosity and lithiophilicity improved the chemical adsorption capacity for lithium polysulfides, thus the polysulfide shuttle was inhibited and the utilization rate of active sulfur material was improved (Figure 5b). During 1000 cycles of charging and discharging at 1 C, 0.052% capacity attenuation and sulfur utilization rate was still 90.7% at 0.1 C [77].

Due to the advantages of being highly conductive and lightweight, etc., carbon materials were further combined with PEDOT: PSS to decorate the separators. For instance, Yi and his colleagues prepared a separator coated with carbon black/PEDOT: PSS (Figure 5c). It could effectively inhibit polysulfides and significantly accelerate Li^+^ migration. When assembled into LSB, the specific capacity was up to 1315 mA h g^−1^ after 1100 charges/discharges at 0.2 C, and the discharge capacity was still up to 699 mA h g^−1^ at the current density of 2 C, indicating the excellent electrochemical performance, which was better than that of the LSB without an interlayer [78]. Lee et al. prepared a PEDOT: PSS/rGO composite as the coating material modified on the separator (Figure 5d). PEDOT: PSS/rGO was well-combined with the separator to physical adsorption and chemical anchor polysulfides to prevent the shuttle effect. After 100 charging/discharging at the rate of 0.5 C, the retained capacity was still as high as 800 mA h g^−1^, which was more than twice that of the LSB without an interlayer [79].

## 5. Conductive Polymer-Based Interlayer Modified Sulfur Electrode

In addition to coating the modified material on the commercial separator and inserting an independent interlayer between the separator and the positive electrode, another effective method to prevent the shuttle of polysulfides is to cover a protective layer on the cathode to reduce the loss of active materials, which is a simple and effective way to reduce the polarization of sulfur cathode, suppress the shuttle effect and improve the specific capacity [32,62,80].

**Figure 6:**
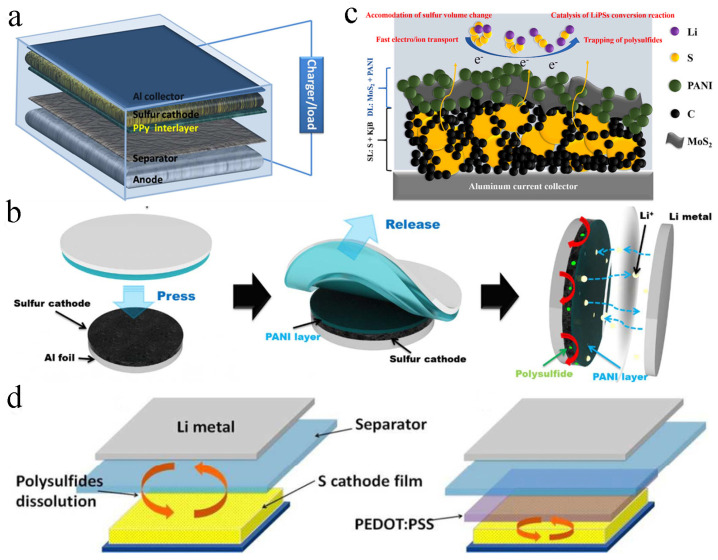
(**a**) Schematic illustration of the functional interlayer in the LSBs. Reproduced from ref. [81] with permission from Elsevier, copyright 2014. (**b**) Schematic representation of double−layer cathode electrode. Reproduced from ref. [34] with permission from IOP (Bristol, UK), copyright 2017. (**c**) Schematic illustration of the PANI−layer coated by a printing method. Reproduced from ref. [84] with permission from Elsevier, copyright 2022. (**d**) Illustration of LSB configuration, lithium−PEDOT: PSS−modified S battery. Reproduced from ref. [85] with permission from The Royal Society of Chemistry, copyright 2015.

Ma and co-workers coated a PPy-functionalized interlayer on the surface of the sulfur cathode, which could suppress the shuttle of polysulfides and improve the utilization of active substances (Figure 6a). When assembled into a battery, the LSB displayed excellent electrochemical performance, with an initial discharge capacity of 719 mA h g^−1^. After 200 charge/discharge cycles at 0.2 C, the retained capacity was up to 846 mA h g^−1^, and the coulomb efficiency was also as high as 94.2% [81]. An oxidative electropolymerization strategy was further developed by Nakamura et al. to prepare a PPy film, and the film was directly coated onto an S/Ketjenblack cathode. The diffusion of polysulfides was suppressed, and the electrochemical performance of LSB was improved [82]. In addition, Gao et al. prepared a spherical PPy interlayer, which prevented polysulfides from passing through the separator. The discharge specific capacity was up to 882 mA h g^−1^ at 1.4 mA cm^−2^ after 100 cycles, with a reversible specific capacity and average coulomb efficiency of 652.7 mA h g^−1^ and 98.1%, respectively [83].

Moon et al. reported that PANI could be covered on the surface of the sulfur electrode by transfer printing; the dissolution of sulfide was significantly inhibited, and the conductivity of the sulfur cathode was significantly improved (Figure 6b). Compared with LSB without interlayers, the capacity retention rate of LSB equipped with printed polyaniline layer modified sulfur cathode was up to 96.4%, and the coulomb efficiency was up to 99.6% after 200 charges/discharges [34]. In addition, Versaci et al. prepared a molybdenum disulfide (MoS_2_)/PANI interlayer (Figure 6c). Both MoS_2_ and PANI acted at the same time, the adsorption capacity was enhanced, the dissolution of polysulfides was inhibited and the kinetic rate of the oxidation-reduction reaction was increased. After charging and discharging 500 times, the specific capacity of the battery assembled with this interlayer was up to 600 mA h g^−1^, 42% higher than the standard sulfur cathode [84]. Li et al. coated the conductive polymer PEDOT: PSS on the surface of the original sulfur electrode (Figure 6d), and the sulfur utilization rate and capacity retention rate were improved. The PEDOT: PSS membrane could suppress the diffusion and migration of polysulfides, maintain the stability of the electrode and provide more active sites to capture and transform polysulfides [85].

## 6. Summary and Prospects

With the development of modern electric vehicles, LSBs with extremely high energy density are attracting increasing attention. However, the commercialization of LSBs still faces the challenge of serious shuttling of polysulfides. Interlayers can alleviate volume expansion and prevent active materials from falling off the electrode. Moreover, it can be clearly seen from Table 1 that the mass loading of interlayers would significantly affect the capacity and cycling stability of the battery. Usually, the electrochemical window of LSBs ranges from 1.7 to 2.8 V (sometimes ranging from 1.5 to 3 V). Capacities and cycling performance of LSBs would be affected by the electrochemical window. Although some progress has been made in the application of conductive polymer-based interlayers, further exploration is still needed to achieve high energy density and stable cycle performance for LSBs. The summary and prospect of each part are as follows:

(1) For the free-standing conductive polymer-based interlayers: The preparation methods mainly include vacuum filtration and vapor deposition. The inhibition of the shuttle effect is the functional groups of conductive polymers and the special structure design of materials. The main problems that confront defects are the high consumption of electrolytes and the high weight of materials, leading to low gravimetric energy density. To solve these problems, the functional interlayer materials need to be lighter and thinner.
(2) For the conductive polymer-based interlayer modified separators: Their preparation methods mainly include vacuum filtration, blade scraping/coating, in situ vapor-phase polymerization, binder-free coating, spray coating, and splash coating. The inhibition of the shuttle of polysulfides is mainly attributed to the functional groups applied on conductive polymers and the special structure design of materials. The main problem conductive polymer-based materials modified separators confront is the poor uniformity of the coating. In situ vapor-phase polymerization and spray coating are the best ways to achieve uniformity of the coating. The adsorption and conversion mechanism of polysulfides needs to be further explored by in situ techniques [86,87,88], such as in situ electron microscopy, in situ FT-IR spectroscopy, etc.
(3) For the conductive polymer-based interlayer modified sulfur electrode: The preparation methods mainly include oxidation polymerization, blade scraping/coating and in situ polymerization. The suppression of the shuttle of polysulfides mainly originates from the increase in functional groups and active sites in conductive polymers. The main problem of the conductive-polymer-based modified sulfur electrodes is the complexity of their preparation. Compared with the coating modification of sulfur electrode, the electrochemical performance of this modification is inferior to the former. In the future, the preparation method needs to be simpler and the interlayer materials need to be multifunctional. The coating needs a stronger adsorption and conversion effect on polysulfides than the method of using a host to load sulfur.

The main limitation of current interlayers is that the preparation methods are relatively complex, and the thickness of the prepared interlayers cannot be completely unified. Adding an interlayer would inhibit the diffusion of lithium ions and the rate performance of the battery. In the future, thinner, lighter and more uniform interlayers with simpler preparation methods should be developed. Further, stronger polysulfides capture ability, and improved capacity and cycling stability are also vital for interlayers. Advanced in situ characterization techniques should be employed to investigate the detailed working mechanisms in order to further modify the interlayer. Finally, the influence of conductive polymer interlayers on the low-temperature performance of LSBs should be carefully evaluated [89,90,91].

## Figures and Tables

**Table 1:** A summary of electrochemical performances of different conductive polymer-based interlayers.

Classification of Conductive Polymer-Based Interlayers	Interlayer Material	Thickness (μm)/Mass Loading (mg cm^−2^)	Conductivity (S cm^−1^)	Initial Capacity (mAh g^−1^) (without Interlayer)	Capacity Increase Rate (%)	Initial capacity (mAh g^−1^) (with Interlayer)	Residual Capacity (mAh g^−1^) (with Interlayer)	Capacity Decay per Cycle (%) (with Interlayer)	Electrochemical Window (V)	Ref.
Free-standing conductive polymer-based interlayers	PPy	35/1	-	-	-	1102 (0.5 C)	712 (0.5 C, 300 cycles)	0.118	1.8–2.8	[24]
	PPy/carbon paper	200/0.1	-	1012.4	9.6	938.8 (0.5 C)	555 (0.5 C, 200 cycles)	0.200	1.7–3	[43]
	PPy/carbon paper	-/1.3	-	777.95	54.8	1204.5 (0.1 C)	853.7 (0.5 C, 300 cycles)	0.097	1.5–3	[44]
	PPy/carbon paper	-/-	-	776	39.8	1085 (0.1 C)	768 (0.1 C, 200 cycles)	0.146	1.5–3	[45]
	PANI/GO	9/2.48	6.62 × 10^−3^	1110	13.6	1261 (0.5 C)	896 (0.5 C, 150 cycles)	0.193	1.7–3	[46]
	PEDOT:PSS/CNT	7/0.7	930	611	50.7	921 (0.5 C)	653 (0.5 C, 200 cycles)	0.145	1.7–3	[36]
Conductive polymer-based interlayer modified separators	PPy nanotube, PPy nanowire	-/1	4.2–4.8	1170 (0.2 C)	2.56	1110.4 (0.5 C)	801.6 (0.5 C, 300 cycles)	0.093	1.8–2.8	[53]
	PPy nanofiber	10/1.4	19.23	1081	14.3	1236 (0.2 C)	1073 (0.2 C, 200 cycles)	0.066	1.7–2.8	[33]
	PPy sphere	8/0.35	-	1100	14.7	1274 (0.2 C)	855 (0.2 C, 100 cycles)	0.329	1.7–2.8	[54]
	PPy/separator/PPy	6.5 × 10^−2^/0.13	3.9 × 10^−4^	1107	14.8	985 (0.1 C)	805 (0.5 C, 250 cycles)	0.083	1.7–2.8	[55]
	PPy/separator/PPy	-/1.8–2 mg	16.7	1038	26	1308 (0.1 A/g)	(1 A/g, 500 cycles)	0.037	1.7–2.8	[56]
	PPy/ZnO	12.4/-	-	930	28.4	1194 (0.2 C)	579 (0.2 C, 100 cycles)	0.515	1.7–2.8	[57]
	PPy/NiCo_2_O_4_	30/0.7	-	790	101	1588 (0.1 C)	423 (2 C, 400 cycles)	0.107	1.7–2.8	[58]
	PPy/graphene	30/0.35	2.4	874	65	1360 (0.2 C)	900 (0.2 C, 200 cycles)	0.169	1.8–2.7	[59]
	Hydrolyzed polyethylene grafted PANI	-/0.92	2.13 × 10^−3^	1221.5	6	1294.8 (0.1 C)	63.4% (1 C, 500 cycles)	0.073	1.6–3	[64]
	PANI nanofiber/MWCNT	8/0.01	-	836	22	1020 (0.2 C)	709 (0.2 C, 100 cycles)	0.305	1.8–2.8	[65]
	SPANI/MWCNT	10/-	-	1047	7.5	1126 (100 mA/g)	913 (100 mA/g, 100 cycles)	0189	1.7–2.8	[66]
	PANI/V_2_O_5_	8/-	-	748	51.4	1132.4 (0.2 C)	586 (1 C, 1000 cycles)	0.037	1.7–2.8	[67]
	PANI/TiO_2_/MWCNT	10/0.4	-	-	-	1220 (0.5 C)	1183 (0.5 C, 100 cycles)	0.030	1.5–2.6	[68]
	PANI/Co-Fe Prussian blue	7/0.2	-	801.05	21.8	975.3 (0.2 C)	603 (1 C, 100 cycles)	0.165	1.7–2.8	[69]
	PEDOT:PSS/Ti_3_C_2_T_X_	49.7/0.8	3.19	866	43.3	1241.4 (0.2 C)	485.3 (0.5 C, 1000 cycles)	0.030	1.7–2.8	[74]
	PEDOT:PSS	-/0.07	10^−6^	-	-	914 (0.25 C)	682 (0.25 C, 1000 cycles)	0.036	1.5–2.8	[76]
	PEDOT:PSS/CTF	0.15/1.4 × 10^−3^	(0.33–0.36) × 10^−3^	970	24.2	1205 (1 C)	577 (1 C, 1000 cycles)	0.052	1.5–3	[77]
	PEDOT:PSS/CB	6.4/0.604	0.045	682	92.8	1315 (0.2 C)	956 (0.2 C, 100 cycles)	0.273	1.5–2.8	[78]
	PEDOT:PSS/rGO	-/0.6	-	834.3	49.8	1249.4 (0.1 C)	812.8 (0.5 C, 100 cycles)	-	1.8–2.8	[79]
Conductive polymer-based interlayer modified sulfur electrodes	PPy	10/0.3	-	940	-	719 (0.2 C)	846 (0.2 C, 200 cycles)	-	1.5–2.8	[81]
	PPy	123/2.3	-	-	-	882 (1.4 mA/cm^2^)	652 (1.4 mA/cm^2^, 100 cycles)	0.261	1.7–2.8	[82]
	Spherical PPy	3.6/-	-	-	-	1037 (4.5 V)	691 (4.5 V, 50 cycles)	0.667	1.5–3	[83]
	PANI	1.5/-	-	1298	-	935 (1 C)	901.34 (1 C, 200 cycles)	0.018	1–3	[34]
	PANI/MoS_2_	200/-	-	1110	11.7	1240 (0.1 C)	440 (0.2 C, 400 cycles)	-	1.7–2.8	[84]
	PEDOT:PSS	4.5wt%	-	867	22.4	1061 (0.2 C)	638 (0.2 C, 100 cycles)	0.399	1.7–3	[85]
